# Is the Anal Component of the Anogenital HPV-Related Disease Overlooked During the Surveillance of Patients Treated for Cervical Intraepithelial Neoplasia?

**DOI:** 10.7759/cureus.44731

**Published:** 2023-09-05

**Authors:** Utku Ozgen, Tolga Guler, Derya Kilic, Ali Gokakin, Muhammed Aykota, Ilknur Kaleli, Yeliz Arman Karakaya, Ugur Sungurtekin

**Affiliations:** 1 Surgery, Pamukkale University, Denizli, TUR; 2 Obstetrics and Gynecology, Pamukkale University, Denizli, TUR; 3 General Surgery, Lokman Hekim Etlik Private Hospital, Ankara, TUR; 4 General Surgery, Pamukkale University, Denizli, TUR; 5 Microbiology, Pamukkale University, Denizli, TUR; 6 Pathology, Pamukkale University, Denizli, TUR

**Keywords:** screening, cervical cancer, cervical intraepithelial neoplasia, anal cancer, human papillomavirus

## Abstract

Aim: To investigate the anal component of the anogenital Human Papillomavirus (HPV) related disease during surveillance of patients treated for cervical intraepithelial neoplasia (CIN).

Methods: Patients were analyzed within two groups according to the histopathological examination of the cervical biopsies: Low-Grade Squamous Intraepithelial Lesion (LSIL) and High-Grade Squamous Intraepithelial Lesion (HSIL) groups. Anal specimens were also collected in the first-year follow-up visit.

Results: All patients had cervical high-risk HPV (HR HPV) infection at admission. At the first-year follow-up, positive HR HPVs were found in 47% of cervical samples. Despite this clearance, the anal HPV infection rate after the first year was 42.5% and 39.6% in LSIL and HSIL groups. Amongst the HSIL group, anal HR HPV positivity was observed in 29.6% of cases without any cervical HPV infection.

Conclusion: A group of women cured of high-grade lesions have ongoing anal HPV infection. It is reasonable to propose that detecting anal HPV could impact the patient's treatment process. Therefore, prospective studies are needed to investigate this group of women's clinical outcomes and define the clearance rate of cervical HPV infection when anal HPV persists.

## Introduction

Anogenital Human papillomavirus (HPV) infection-related neoplasias are considered as sexually transmitted diseases [1,2]. Women with cervical dysplasia carry a high risk for anal intraepithelial neoplasia and anal cancer [3]. This is because of the high incidence of anal infection with oncogenic HPV types within these women [4]. Anal infection with oncogenic HPV genotypes is also more common in women with high-risk cervical HPV infection [5]. The clinical significance of this concomitant cervical-anal infection is still unknown.

The time interval between the diagnosis of cervical dysplasia and the onset of anal cancer generally takes a relatively long period, up to a decade or more [6]. Nevertheless, there is a lack of evidence regarding the impact of cervical treatment on the progression of the disease in the anal canal. Hence, there is a necessity for prospective studies to explore whether the treatment of cervical dysplasia affects the onset and progression of anal cancer. This long pre-invasive period allows carrying out screening tests. However, Santoso et al. documented that anal cytology has a poor sensitivity (8%) in diagnosing anal intraepithelial neoplasia in women with cervical dysplasia [7]. On the other hand, HPV-related biomarkers showed promising results for detecting precancerous anal lesions in HIV-positive men [8]. Since HPV-based screening is efficient in cervical cancer screening [9], suggesting a similar approach for anal cancer in selected high-risk populations may seem reasonable. However, there is a paucity of data regarding the clinical value of anal HPV testing after cervical intraepithelial neoplasia (CIN) diagnosis in women.

The current explanation for the development of cervical neoplasms states a step-by-step development, starting with a persistent high-risk HPV infection, followed by the development of high-grade lesions, resulting in invasive cancer [10]. In women with cervical intraepithelial neoplasia (CIN) history, cervical HPV infection may resolve. However, recurrent infection is always possible, even with the same HPV genotype [11]. This recurrent infection may further progress into an invasive disease. Jenkins et al. argued that dormant HPV presence in the basal epithelial layer or re-inoculation may be the reason for this recurrent HPV infection [12]. Apart from inoculation via sexual contact, autoinoculation might also explain re-inoculation. An autoinoculation mechanism was proposed for beta-HPVs; however, there is no prospective data for alpha-HPVs [13]. Due to its proximity to the vagina, the most likely origin of autoinoculation to the cervix could be the anal canal. By this, Lammé et al. pointed out that concomitant anal HPV infection may increase the risk of reinfection in the cervix, which may progress to high-grade lesions [5]. The clinical value of anal HPV testing is currently undetermined for the prognosis of cervical dysplasia and development of subsequent anal intraepithelial lesions. Investigating anal HPV infection during surveillance is needed to uncover the role of the anal component of this anogenital HPV-related disease. This study investigates the existence and co-occurrence of anal and cervical HPV infection among women recruited from an HPV-based cervical cancer screening program and treated with excisional procedures for high-grade lesions or followed for low-grade lesions.

## Materials and methods

Study population

This study was conducted at a single Cervical Disease Screening and Treatment Unit center. The Institutional Ethics Review Board approved the study. The study group was composed of women aged between 30-65 years. The inclusion criteria for the study group were as follows: women with a positive cervical HR HPV test before admission to colposcopy, women older than 30 years of age, histological diagnosis of Low-Grade Squamous Intraepithelial Lesion (LSIL) or High-Grade Squamous Intraepithelial Lesion (HSIL), conservative management if LSIL was diagnosed. If the initial histopathological diagnosis was HSIL, a standard cervical excision procedure by the same surgeons (TG and DK) was performed. Women without a histological diagnosis were excluded from the analysis. Other exclusion criteria included: history of any cervical therapeutic intervention before initial colposcopy, history of other cervical or genital dysplasia, known immunosuppressive disorder, being under immunosuppressive medication, undergoing ablative treatment for CIN, previous diagnosis of any cancer, history of vaccination against HPV. The participant's age, the number of sexual partners, age of sexual debut, parity status, smoking status, former or current oral contraceptive use, and other related patient data (HPV genotypes at admission and first-year follow-up) were recorded on a data sheet. Patients were analyzed within two groups according to histopathological examination of the cervical biopsies: LSIL and HSIL groups.

Specimen collection and HPV genotyping procedure

Anal specimens were collected in the first-year follow-up visit by inviting the patients to participate in the study. Informed consent was obtained from women willing to participate in anal HPV testing. Anal specimens were collected by inserting Dacron swabs into the anal canal and gently rotating them in a circular motion. Those swabs were placed into liquid transport media and transported to the microbiology laboratory. A technician blinded to the subject's medical history performed all laboratory procedures. According to manufacturer instructions, DNA extraction was performed using the "EZ1® Advanced XL Nucleic Acid Purification" instrument (Qiagen Inc., Valencia, CA). Amplification and detection were carried out using the "HPV Genotypes 14 Real-TM Quant" kit (NLM, Settala MI, Italy) that allowed the identification of 14 high-risk genotypes (16, 18, 31, 33, 35, 39, 45, 51, 52, 56, 58, 59, 66 and 68). Anal swabs were reobtained if the first sample was inadequate for analysis.

Statistical analysis

Statistical tests were performed by PSPP 1.0.1 and R (with EasyR plugin) software. The Shapiro-Wilk test examined the continuous variables with normal and abnormal distributions, while the parametrical t-test was used for the normally distributed continuous variables. When applicable, the nominal variables were analyzed using Pearson's chi-square or Fisher's exact test. The continuous variables were presented using the mean±standard deviation (SD), and the categorical variables were presented as the number of cases and their percentage. The Bonferroni adjustment was performed for all possible multiple comparisons to control for type I errors. A p-value less than 0.05 (<0.05) was set as statistically significant.

## Results

Three hundred one patients underwent colposcopic investigation after primary HPV screening. During surveillance, 91 were lost to follow-up in the first year visit. After excluding the ones according to the abovementioned criteria, 83 women who met the inclusion criteria and had been managed with the diagnosis of CIN were included in the study. All had previously undergone colposcopy and cervical biopsy. Biopsy-proven HSIL cases had been treated with an excisional procedure. There was a total of 40 women with LSIL diagnosis and 43 women with HSIL lesions on their follow-up who agreed to participate in the present study. These were defined as the LSIL and HSIL groups. All participants had undergone anal sampling for HPV testing during their first-year follow-up visit. The characteristics of the patients are presented in Table 1.

**Table 1:** Patients characteristics and risk factors associated with cervical intraepithelial neoplasia. LSIL, low-grade squamous intraepithelial lesion; HSIL, high-grade squamous intraepithelial lesion; BIM, body mass index; COC, combined oral contraceptive; HRT, hormone replacement therapy; IUD, intrauterine device

	LSIL Group (n; %) n=40	HSIL Group (n; %) n=43	Overall (n; %) n=83	p-value
Physical parameters				
Mean age (SD, minimum: maximum)	43.6 (8.7; 31: 64)	43.4 (9.2; 31: 62)	43.5 (8.9; 31: 64)	0.946
BMI (SD, minimum: maximum)	26.8 (4.5, 17.9: 38.5)	27.1 (5.0, 20.7: 41.1)	26.9 (4.8, 17.9: 41.1)	0.784
Reproductive and sexual history				
Number of pregnancies (SD, minimum: maximum)	2.8 (1.7, 0: 9)	2.5 (1.1, 0: 5)	2.7 (1.4; 0: 9)	0.348
Number of births (SD, minimum: maximum)	1.9 (0.9, 0: 4)	2.0 (1.0, 0: 5)	2.0 (0.9, 0: 5)	0.818
First coital age (SD, minimum: maximum)	19.6 (3.8; 15: 35)	21.6 (4.2, 14: 31)	20.7 (4.1, 14: 35)	0.035
Coital age <18	17 (42.5%)	10 (23.3%)	27 (32.5%)	0.061
Number of the sexual partner >1	9 (22.5%)	8 (18.6%)	17 (20.5%)	0.660
Smoking status				
Never smoked	21 (52.5%)	29 (67.4%)	50 (60.2%)	0.185
Active smoking (>3 cigarettes/day in the last year)	10 (25%)	9 (20.9%)	19 (22.9%)	0.795
Smoke exposure (active+passive smoking in the last year)	16 (40%)	18 (41.9%)	34(41%)	0.863
Contraception and/or HRT				
Never used	32 (80%)	29 (67.4%)	61 (73.5%)	0.195
COC and/or HRT user	3 (7.5%)	6 (14%)	9 (10.8%)	
IUD user	5 (12.5%)	4 (9.3%)	9 (10.8%)	
Medical history				
Diabetes mellitus	1 (2.5%)	8 (18.6%)	9 (10.8%)	0.019
Autoimmune disease	3 (7.5%)	6 (14%)	9 (10.8%)	0.485

Cervical and anal HPV infection status during the first-year follow-up visit was summarized in Table 2. A total of 3 samples were regarded as inadequate. None of them remained inadequate for analysis after sequential anal swab collection. Overall, positive HR HPVs were found in 47% and 41% of cervical and anal samples, respectively. Cervical and anal HPV infection prevalence was comparable between LSIL and HSIL cases (Table 2).

**Table 2:** Cervical and anal HPV infection status after the first year from the initial diagnosis. LSIL, low-grade squamous intraepithelial lesion; HSIL, high-grade squamous intraepithelial lesion

Parameters	LSIL (n; %) n=40	HSIL n; %) n=43	Overall ( n; %) n=83	p-value
Cervical HPV infection				0.159
16/18 positive HPV infection	9 (22.5%)	5 (11.6%)	14 (16.9%)	
16/18 negative HPV infection	14 (35.0%)	11 (25.6%)	25 (30.1%)	
No HPV infection	17 (42.5%)	27 (62.8%)	44 (53.0%)	
Anal HPV infection				0.760
16/18 positive HPV infection	8 (20.0%)	10 (23.3%)	18 (21.7%)	
16/18 negative HPV infection	9 (22.5%)	7 (16.3%)	16 (19.3%)	
No HPV infection	23 (57.5%)	26 (60.5%)	49 (59%)	

**Figure 1:**
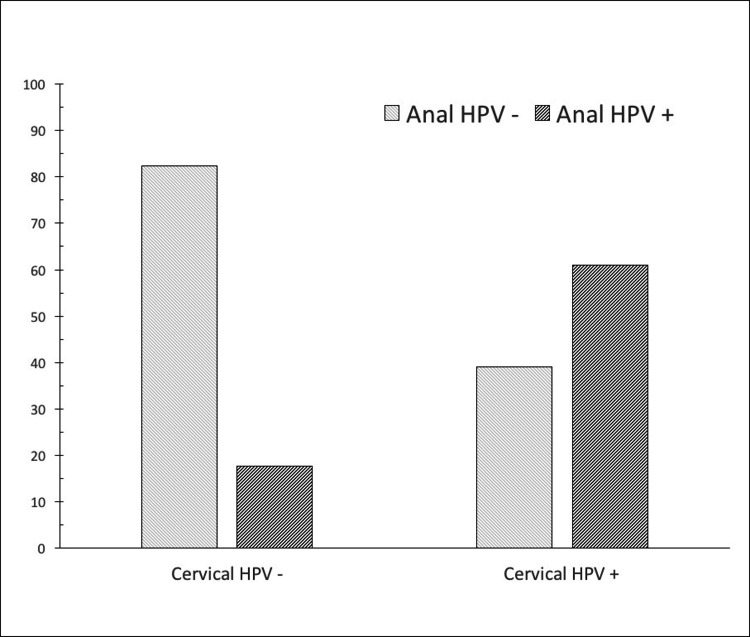
HPV infection status in the anal canal concerning cervical HPV infection. Among LSIL cases, active HPV infection of the anal canal was seen in 60.9% of cervical HPV-positive cases and 17.6% of cervical HPV-negative cases, p=0.006). HPV: Human Papillomavirus; LSIL: Low-Grade Squamous Intraepithelial Lesion

**Figure 2:**
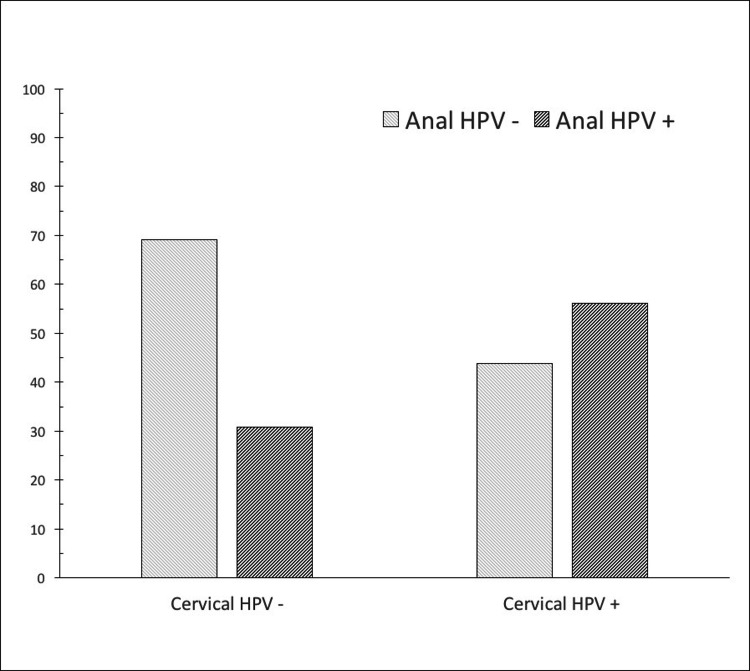
HPV infection status in the anal canal with respect to cervical HPV infection. Among HSIL cases, active HPV infection of the anal canal was seen in 56.2% of cervical HPV-positive cases and 29.6% of cervical HPV-negative cases, p=0.084). Among HSIL cases, active HPV infection of the anal canal was seen in 56.2% of cervical HPV-positive cases and 29.6% of cervical HPV-negative cases, p=0.084). HSIL: High-Grade Squamous Intraepithelial Lesion; HPV: Human Papillomavirus

In the cervical samples of the HSIL group, type 16 and/or 18 were found in 11.6% and other high-risk types in 25.6% of the cases. On the other hand, in the anal samples of the same HSIL group type, 16/18 was determined in 23.3% of the cases. HPV infection status in the anal canal was also analyzed with respect to current cervical HPV infection (Figure 1-2).

In the LSIL group, the rate of anal HPV infection was higher in positive cervical HPV cases than in negative cervical HPV cases (60.9% vs. 17.6%, respectively, p=0.006). A similar trend was observed in the HSIL group, as active anal HPV infection rates in cervical HPV positive and negative cases were 56.2% vs. 29.6%, respectively. However, this difference did not reach statistical significance (p=0.084).

 In the HSIL group, HPV genotype 16 and/or 18 persisted only in 5 cases (11.6%) and cervical HPV infection was cleared in 27 (62.8%) women. Among these women with HPV clearance, 29.6% were found to be positive for HR HPV DNA in their anal canal (Table 3).

**Table 3:** HPV 16/18 infection status at the cervix and the anal canal in patients treated for HSIL. LSIL, low-grade squamous intraepithelial lesion; HSIL, high-grade squamous intraepithelial lesion; HPV: Human Papillomavirus

		Cervical HPV results in HSIL group (n=43)		
		HPV negative n=27 (62.8%)	16/18 negative HPV infection n=11 (25.6%)	16/18 positive HPV infection n=5 (11.6%)
Anal HPV infection	HPV negative n=26 (60.5%)	19 (70.4%)	5 (45.5%)	2 (40%)
	16/18 negative HPV infection n=7 (16.3%)	2 (7.4%)	4 (36.4%)	1 (20%)
	16/18 positive HPV infection n=10 (23.3%)	6 (22.2%)	2 (18.2%)	2 (40%)
	Total n=43 (100%)	27 (100%)	11 (100%)	5 (100%)

Moreover, 75% of this anal HPV infection was with HPV genotype 16 and/or 18. Overall in the HSIL group, 39.6% of women were found to carry HR HPV DNA in their anal canal after the first year. On the other hand, in the LSIL group, clearance of cervical HPV infection was lower (42.5%) (Table 4).

**Table 4:** HPV 16/18 infection status at the cervix and anal canal in patients followed for LSIL. LSIL, low-grade squamous intraepithelial lesion; HSIL, high-grade squamous intraepithelial lesion; HPV: Human Papillomavirus

		Cervical HPV results in LSIL group (n=40)		
		No HPV infection n=17 (42.5%)	16/18 negative HPV infection n=14 (35.0%)	16/18 positive HPV infection n=9 (22.5%)
Anal HPV infection	No HPV infection n= 23 (57.5%)	14 (82.4%)	6 (42.9%)	3 (33.3%)
	16/18 negative HPV infection n= 9 (22.5%)	2 (11.8%)	6 (42.9%)	1 (11.1%)
	16/18 positive HPV infection n= 8 (20.0%)	1 (5.9%)	2 (14.3%)	5 (55.6%)
	Total n=40 (100%)	17 (100%)	14 (100%)	9 (100%)

Among this HPV-cleared group, only one (5.9%) woman was found to have genotype 16 and/or 18 in her anal swab. However, amongst 9 women who were found to be positive for genotype 16 and/or 18 in their cervical swabs, 5 (55.6%) of these women were detected to carry genotype 16 and/or 18 in the anal canal.

## Discussion

This current study analyzed cervical and anal HPV infection rates among women managed for LSIL and HSIL of the cervix. All women had initially undergone colposcopy, and those with histologically proven HSIL were treated with an excisional procedure (cervical conization). At the first-year follow-up, positive HR HPVs were found in 47% and 41% of cervical and anal samples, respectively. Cervical infection prevalence was 57.5% and 37.2% among LSIL and HSIL groups, indicating excisional procedures in women with HSIL resulted in complete HPV clearance in more than half of the cases. Despite this clearance, this study demonstrated that the anal HPV infection rate after the first year was 42.5% and 39.6% among LSIL and HSIL cases. In women with HSIL, type 16 and/or 18 was found in 11.6% of the cervical samples. However, type 16/18 took a more dominant place in the anal samples of the same women, with a prevalence of 23.3%.

Moreover, amongst the HSIL group, anal HR HPV positivity was observed in 29.6% of cases without any cervical HPV infection. Surprisingly, 22.2% of women who were negative for cervical HPV were found to be positive for HPV 16/18 in their anal swabs. Anal HPV 16/18 persisted in 22.2% of the HSIL cases even after a complete cervical HPV clearance. 

Several previous studies have investigated coexisting anal HPV colonization and cervical HPV infection. Guler et al. [14] reported a relatively higher prevalence (51.9%) of coexisting anal HPV infection than this study found that most of the women with anal HPV infection were infected with oncogenic or probable oncogenic types (64.6 %) with a 58.3% rate of partial concordance between the two sites. Sehnal et al. investigated two groups of women (high-risk groups: high-grade CIN and low-risk groups: non-neoplastic or CIN1) concerning HPV infection in cervical (n=269) and anal (n=261) samples [15]. Overall, in the high-risk group, it was reported that 55 cases (21.1%) were detected with HR HPV. These results are lower than the present study findings of 42.5% and 39.6% in LSIL and HSIL patients, respectively. The possible reason for this discrepancy between research findings could be that women in the current study were initially referred with a diagnosis of cervical HR HPV infections from an HPV-based screening program. This implies that clinical findings and anal HPV status of patients with high-risk cervical HPV infections differ according to inclusion criteria.

Another study investigating anal HPV prevalence in women attending a colposcopy clinic found the presence of HPV in the anus to be 56.3% [4]. This is conclusive to the results found within the present research. It also reported a relatively lower anal sexual contact history (16.9%). However, that study population was heterogeneous; 47 patients were on their first check-up after treatment (15 LEEP, 2 conizations), and 67 patients had an older lesion analyzed afterward without a defined time interval (39 LEEP and 1 conization). Nevertheless, the authors documented the same results found within this study; a considerably high number of women with negative cervical HPV tests had positive anal samples. A previous report presented the prevalence of anal HPV infection in women with CIN, VAIN, or VIN, which are known to be related to HPV infection [16]. The researchers documented a high prevalence of anal HPV infection in their high-risk population (51%), a little higher than the anal HPV positivity rate of 39.6% after treatment of HSILs found within this study. The most probable explanation for this result is the possibility that anal HPV infection may be cleared slower than cervical HPV infection since no therapeutic interventions were applied to the anal canal within this study. However, there has been no prospective data in the literature investigating the long-term clearance rate of anal HPV infection after treatment for cervical HSIL.

The widespread HPV-based screening would result in an ever-increasing number of women with high-risk HPV infection with normal cervical cytology results [17]. One value of this analysis comes from its position representing real-world data regarding the surveillance results of women managed after a nationwide screening program. A homogeneous group of women were analyzed in this current study. This study is thought to be the first to assess anal HPV status among women referred and managed after an HPV-based screening program. Another strength of this study was the standardized management protocol used in all women after initial colposcopy. However, one limitation of the present study is the lack of data regarding the initial anal HPV status of the LSIL and HSIL cases.

This study did not include a long-term prognosis that precludes determining the sensitivity and specificity of anal HPV testing on the recurrence of cervical HPV infection and related dysplasia. Another limitation of this study is the lack of matching for diabetes mellitus status between the LSIL and the HSIL group. Since glucose status is important in intraepithelial lesions, there is a need to prospectively assess the clinical value of this issue in the development and association of anal HPV infection in LSIL and HSIL groups.

## Conclusions

In conclusion, this study found that women who have been categorized as being cured for high-grade lesions have been found to have ongoing anal HPV infection (including HPV types 16 and/or 18). This anal HPV infection is observed even in negative cervical HPV cases. Longitudinal studies are needed to define the value of anal HPV testing during anogenital HPV-related disease.
